# Marine Origin Collagens and Its Potential Applications

**DOI:** 10.3390/md12125881

**Published:** 2014-12-05

**Authors:** Tiago H. Silva, Joana Moreira-Silva, Ana L. P. Marques, Alberta Domingues, Yves Bayon, Rui L. Reis

**Affiliations:** 1 3B’s Research Group—Biomaterials, Biodegradables and Biomimetics, University of Minho, Headquarters of the European Institute of Excellence on Tissue Engineering and Regenerative Medicine, AvePark, 4806-909 Taipas, Guimarães, Portugal; E-Mails: joana.silva@dep.uminho.pt (J.M.-S.); ana.marques@dep.uminho.pt (A.L.P.M.); albertadomingues@gmail.com (A.D.); rgreis@dep.uminho.pt (R.L.R.); 2 ICVS/3B’s—PT Government Associate Laboratory, Braga/Guimarães, Portugal; 3 Covidien—Sofradim Production, F-01600 Trevoux, France; E-Mail: yves.bayon@covidien.com

**Keywords:** collagen, marine biotechnology, marine biomaterials, tissue engineering, biomedical application, valorization, marine byproducts

## Abstract

Collagens are the most abundant high molecular weight proteins in both invertebrate and vertebrate organisms, including mammals, and possess mainly a structural role, existing different types according with their specific organization in distinct tissues. From this, they have been elected as one of the key biological materials in tissue regeneration approaches. Also, industry is constantly searching for new natural sources of collagen and upgraded methodologies for their production. The most common sources are from bovine and porcine origin, but other ways are making their route, such as recombinant production, but also extraction from marine organisms like fish. Different organisms have been proposed and explored for collagen extraction, allowing the sustainable production of different types of collagens, with properties depending on the kind of organism (and their natural environment) and extraction methodology. Such variety of collagen properties has been further investigated in different ways to render a wide range of applications. The present review aims to shed some light on the contribution of marine collagens for the scientific and technological development of this sector, stressing the opportunities and challenges that they are and most probably will be facing to assume a role as an alternative source for industrial exploitation.

## 1. Introduction

Collagen is a general term to define a group of structural proteins of the extracellular matrix, organized in a fibrillar arrangement [1,2]. Collagens display a palette of unique and numerous features, largely and advantageously exploited in the food, medical devices and pharma industries. They are ubiquitously found in living beings, in quite conserved forms, both in terms of gene and amino acid sequences, especially in the triple helix structure. They can also be very abundant, particularly in mammals (up to 25% of the total proteins) as structural and biologically active components of tissues including skin, bone and cartilage [3]. It has a complex structural and hierarchical organization, with more than 20 types of collagens reported up to now [4]. The different types of collagens are differently distributed in animal tissues. The following list gives examples of tissues where collagen types are the most abundant: Type I—bone, dermis, tendon, ligaments, cornea; Type II—cartilage, vitreous body, nucleus pulposus; Type III—skin, vessel wall, reticular fibers of most tissues (lungs, liver, spleen, *etc*.); Type IV—basement membranes, Type V—often co-distributes with Type I collagen, especially in the cornea. This naturally favored the commercial exploitation of the standard abundant collagens (collagens I–V), by isolating and purifying them, mostly from human, bovine and porcine tissues, by conventional, high yield manufacturing processes, leading to high quality collagen batches [5]. Generally, collagens are formed by polypeptide chains constituted by repeating triplets Gly-X-Y of Glycine and two other amino acids, where proline and hydroxyproline (Hyp) are the most common, of about 1000 total amino acids [6,7,8,9]. In some cases, like in *H. sieboli* glass sponge, X and Y positions can be both occupied with isomers of Hyp: 3-Hyp and 4-Hyp, an unusual double hydroxylation. In this sponge, this hydroxylated collagen plays an important role in biosilicification and, specifically trans-3-Hyp in collagen is a key on the stabilization of silic acid molecules and in the precipitation of silica (mineralization). The silica network of living organisms at early evolutionary stages was template by hydroxylated collagen [10,11]. Collagen polypeptide chains are structurally organized in three α-helices forming its secondary structure. This is called tropocollagen, a protein consisting of three polypeptides units [9]. Mammal collagen I typically results of the association of two α_1_(I) and one α_2_(I) chains. In other collagen types the three α-helices are distinct, as for instance in collagen IX [2,4] or can be all the same such as in collagen III. The polypeptide chains wrap around one another forming a characteristic triple helix-tertiary structure. As in all protein complexes, the organization of several protein molecules—quaternary structure—is determinant of protein function and in collagen the triple helices self-assemble in staggered formation to form collagen fibrils. Those fibrils are also packed together to form collagen fibers, responsible for the tensile strength of this material [4,12].

The structural organization of collagen molecules can be lost by a process called denaturation, an irreversible kinetic process, resulting in random coiled polymeric chains, termed gelatin. Denaturation can be promoted by chemical treatments or simply by thermal treatment, when the helix-coil transition temperature is exceeded [13].

The thermal denaturation of collagen is historically related with its application. In fact, collagen was formerly known as the component of connective tissue that renders gelatin upon boiling and that process was used to produce glue [1]. Indeed, its use as glue was already known about 8000 years ago, near the Dead Sea, and is probably the oldest known glue [14].

Nowadays, collagen has a wide range of applications in the health-related sectors, namely in cosmetics, the pharmaceutical industry and in medical care (including plastic surgery, orthopedics, ophthalmology and dentistry) [15]. In non-health sectors, a noteworthy use of collagen is in the food sector (food processing, as additive and nutraceuticals), but most often as gelatin, *i.e.*, in its denatured form [16].

**Figure 1:**
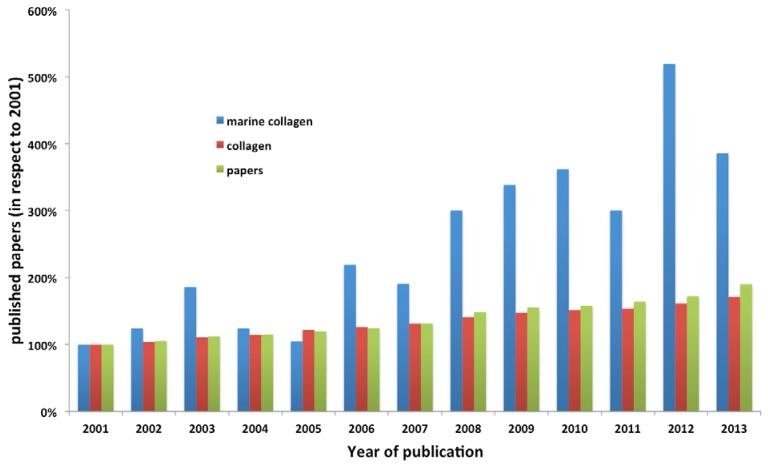
Increment on the number of papers on collagen, marine collagen and in total, published in the last 13 years (XXI century), taking 2001 as reference. The data was collected from search in ISI Web of Knowledge™ using the terms collagen and marine collagen.

The high potential of collagen use has been the rational for intense research on collagen applications over the years. On Figure 1, one can observe the impressive and continuous increase in the number of papers on collagen published in this 21st century (data collected from search in ISI Web of Knowledge™ using the terms “collagen” and “marine collagen”, the total number of papers, taking 2001 as reference), from about 15 thousand in 2001 to more than 26 thousand in 2013, experiencing the same increment rate of the total number of papers.

Regarding its industrial exploitation, collagen has mainly bovine and porcine origins, which have been a matter of concern in the last years. In fact, due to religious constraints related with avoidance of porcine and bovine products and to the recent episode of the wide scale bovine spongiform encephalopathy (BSE) outbreak in bovines, other collagen sources are being debated. In this regard, besides the use of recombinant technology (more expensive and not always effective), the use of collagen with marine origin is being considered highly attractive by the industry as an important alternative source [17,18]. There is also significant research on marine origin collagens, as can be seen by the number of papers published in the last years on this subject depicted in Figure 1. Indeed, it is highly motivating to observe that while the number of published papers on collagen is increasing in the same rate of the total number of published papers (showing that collagen is an active field of research), the number of published papers on marine collagen is experiencing an higher rate, indicating the growing attraction of such material in the last years.

On the present review paper, we looked into this research, commenting on the work that has been performed regarding collagen extraction from different marine resources and its further use in biomedical applications, and shedding some light on future perspectives for this material.

## 2. Marine Collagens

### 2.1. Sources and Isolation

Marine collagens can be obtained from different sources. Several studies have been focusing on marine collagens, namely on its extraction from different sources, such as fishes, or invertebrate marine animals, such as marine sponges or jellyfish (Table 1).

A considerable amount of fish weight (about 75%) is discarded, in the form of skins, bones, fins, heads, guts and scales. From these residues, it is possible to obtain collagen, with an important increase of the economic value of the by-products [19]. It is obtained predominantly type I collagen from skin, tendon, bone and muscle (epimysium), which is the most abundant type of collagen. Nevertheless, type II collagen can also be obtained if fish cartilage is the selected source to work on.

Solubility of collagen is age dependent: collagenous tissues of older animals have a higher number of crosslinkers, which makes them harder to solubilize than collagenous tissues from young animals [20]. Also, fish that undergo a poor diet (starving fish) produce more collagen than well-fed fish. Depending on the collagen source, different techniques have been proposed to obtain collagen-based macromolecules. Still, it is possible to define a general methodology to isolate collagen from fish by-products and other marine sources, composed by three important steps: preparation, extraction and recovery.

Preparation of fish parts involves cleaning, separation of animal parts, size reduction by cutting or mincing the samples and a chemical pre-treatment to remove non-collagenous proteins, pigments or fats. In jellyfish for example, it is common to separate the arms from the umbrella and, this last one, in mesoglea, exumbrella and subumbrella [21]. In the case of fish, they are split up in skins, scales, fins and fish bones, because its composition is different (e.g., mineralization in fish bones and scales) and so the applied methodology to extract collagen must have other preparation steps. Size reduction of these compounds is also important to facilitate acid action. The common method to remove non-collagenous proteins is the use of sodium hydroxide (NaHO). The effectiveness of the removal is dependent on time, temperature and on the concentration of NaHO solution. Sadowska *et al.* [22] also proposed the use of sodium chloride (NaCl), besides NaHO, to remove non-collagenous proteins from codfish skin. However, the NaCl solution demonstrated lower efficacy of removing albumins and globulins, when compared with NaHO. The removal of fats and pigments can be achieved by the use of alcohols (namely butyl-alcohol or ethanol) and oxygen peroxide, respectively [19,23,24,25,26,27]. For bone, cartilage and scales samples, the use of ethylenediaminetetraacetic acid (EDTA) is recommended for demineralization purposes, due to its chelating action over calcium ions, which will render substrates with greater surface area for a better collagen extraction [26,28,29,30]. Alternatively, 1.0 M HCl can also be used for demineralization purposes, at ratios of about 1:3 (w/v) [31].

**Table 1:** Examples of the different types of marine collagens that have been isolated and the variety of marine sources that have been used for such purpose.

Collagen Type	Source of Collagen	Source Tissue	Yield	References
**Type I**	Bigeye snapper	Bone	ASC: 1.59%	[28]
		Skin	ASC: 10.94%	
	Largefin longbarbel catfish		ASC: 16.8%; PSC: 28.0%	[23]
	Seaweed pipefish		ASC: 5.5%; PSC: 33.2%	[32]
	Brown backed toadfish		PSC: 54.3%	[19]
	Ocellate pufferfish		ASC: 10.7%; PSC: 44.7%	[24]
	Lizard fish	Scales	ASC: 0.79%	[33]
	Horse mackerel		ASC: 1.51%	
	Grey mullet		ASC: 0.43%	
	Flying fish		ASC: 0.72%	
	Yellowback seabream		ASC: 0.90%	
	Bigeye tuna	Bone	-	[34]
	Squid	Skin	53%	[7]
	Cuttlefish	Skin	ASC: 0.58%; PSC: 16.23%	[35]
	Edible Jellyfish	Umbrella	46.4%	[36]
**Type II**	Brownbanded bamboo shark	Cartilage	ASC: 1.27%; PSC: 9.59%	[29]
	Blacktip shark	Cartilage	ASC: 1.04%; PSC: 10.30%	
	Ribbon jellyfish	Umbrella	PSC: 9%–19%	[21]
**Type IV**	Marine Sponge		30%	[37,38,39]

For the extraction phase, an acid solution is widely used for the solubilization of collagen, which is further denominated Acid Soluble Collagen (ASC). Skierka and Sadowska [40] tested the influence of different acids on collagen extraction from codfish skin, including hydrochloric, citric, acetic and lactic acids, from which acetic and lactic acids revealed to be the ones rendering higher collagen extraction yields. However, the collagen extraction process normally returns a low yield. To overcome this, researchers have been applying an enzymatic treatment by using proteolytic enzymes non-specific for collagen, to help in the solubilization process, such as trypsin, pancreatin, ficin, bromelain, papain or pepsin, the last one being the most used [41,42]. By applying pepsin, the resulting extract is named Pepsin Soluble Collagen (PSC) or atelo-collagen. This treatment is very useful, since it cleaves peptides specifically in telopeptide region of collagen, which are non-helical ends, and thus, by hydrolyzing some non-collagenous proteins, increases the purity of collagen. It results in a much more efficient collagen extraction, since it makes the sample ready to solubilize while reducing, at the same time, the antigenicity caused by telopeptides [43,44]. For this reason, it is common to use this proteolytic procedure after the extraction of ASC, obtaining, thus, the abovementioned PSC. Antigenicity of collagen is not only derived from its telopeptides, being also related to the presence of noncollagenous proteins, cells and cell remnants [45], being the above-mentioned method of NaHO treatment of raw materials important for the removal of this source of antigenicity.

For the recovery step, collagen needs to be precipitated, generally achieved by adding NaCl to a final concentration that can vary between 2.3 M and 2.6 M in Tris-HCl (pH 7.5). Resultant precipitate is collected by centrifugation, dissolved in 0.5 M acetic acid,, dialyzed and freeze dried, thus acquiring a dry acid soluble and pepsin soluble collagen [19,24,25,26,28,29,32,42,46,47,48].

From jellyfish (the large portion of the body is called umbrella, that is divided in a major component called mesoglea and in outer skins: exumbrella and subumbrella [11]), collagens are obtained from mesoglea, following a methodology based on solubilization in acetic acid solution, typically during three days. Extracts are then dialyzed against Na_2_HPO_4_ solution, resulting in precipitated collagens, which can be separated by centrifugation. The produced collagens can be then purified by a re-precipitation method: solubilization in acetic acid and precipitation by addition of solid NaCl. ASC can also be submitted to pepsin digestion, to obtain atelo-collagen [49,50].

When considering marine sponges, the available methodologies are different, since sponge collagens are not soluble in acid solutions. Different methods can be found, but Swatschek *et al.* [39] proposed a particular procedure aiming to scale-up the *Chondrosia reniformis* sponge collagen production, based in a barely basic solution together with a chaotropic agent. That procedure consisted on the treatment of sponges with 100 mM Tris-HCl buffer (pH 9, 10 mM EDTA, 8 M Urea, 100 mM 2-mercaptoethanol), during 24 h, at room temperature. Sponge collagens were solubilized, separated by centrifugation and further precipitated by decreasing pH to 4 with addition of acetic acid. 

### 2.2. Characterization Methods

The characteristics of collagens *in vivo* are unique and when the goal is to mimic the collagen role in extracellular matrix, as is the case of tissue engineering approaches producing collagen-based matrices for cell culture, it is of extremely importance the evaluation of both native and processed collagen properties. Since there are so many sources from where collagens can be extracted, and so many types, it is crucial to study its characteristics with particular care and accuracy.

There are several techniques that can be used to characterize collagen samples (in solution or in solid state), addressing structural, chemical and morphological features. With a good characterization and, consequently, a better knowledge of the sample, it is easier to have a desired biological response or to establish a relationship between results and features of the produced structure [51]. The goal is to continuously improve the results, by trying to control the sample features, resulting in a more predictable and accurate biological response.

**Figure 2:**
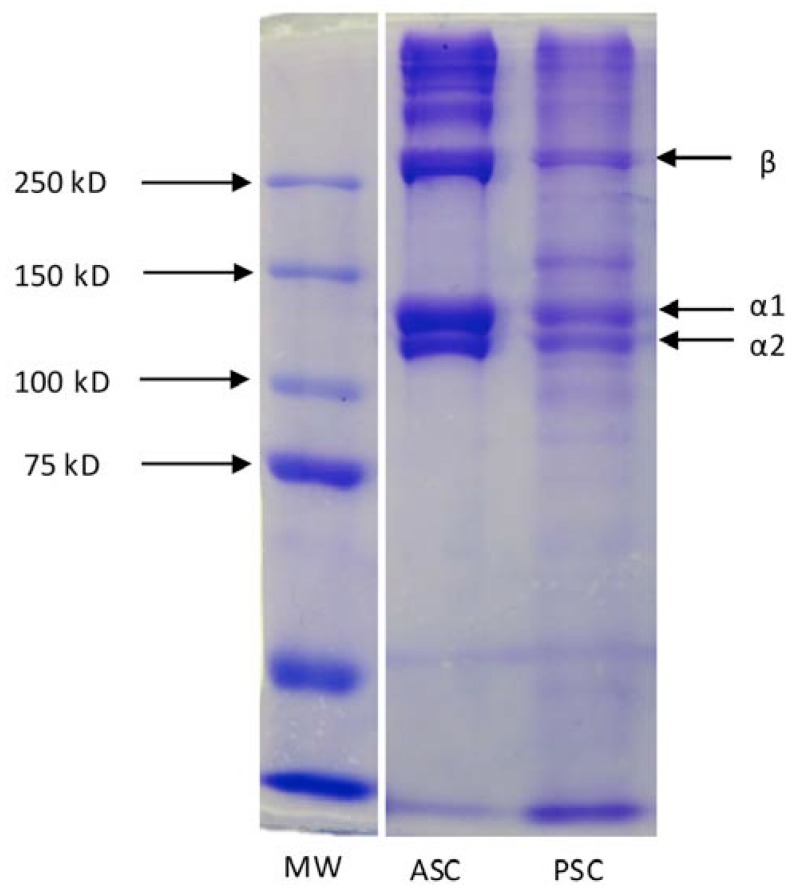
SDS-PAGE pattern of collagen extracted from the skin of codfish *Gadus morhua* by acetic acid treatment (ASC) and in the presence of pepsin (PSC).

One of the methods that are used to characterize collagen purity and breakdown is the Sodium dodecyl sulfate polyacrylamide gel electrophoresis (SDS-PAGE). With gel electrophoresis, proteins and their fragments can be separated, based on their size. It is possible to observe the protein fragments by loading protein samples in the small wells of the gel, which, under an applied electrical field, travel through the gel matrix depending on their size: the smallest go further than the large ones, which stay trapped in the gel net. From this, it is possible to compare with collagens obtained from other sources, taken as reference, and propose an identification of the collagen type (I, II, …) when the collagen bands are similar. In the case of collagens from the same type but from different species, where the amino acid sequence is changed although the same chain types are present (for instance, α1, α2 and β chains in type I collagen), slightly shifts in the position of the bands may be observed [52]. This is most probably caused by small differences in the molecular weight as a consequence of different amino acid sequences. In Figure 2 it is shown a SDS-PAGE pattern of ASC and PSC extracted from the skins of codfish.

It is also common to quantify the content of hydroxyproline, since this amino acid is almost exclusive to collagens, being present in insignificant amounts in other proteins. The content of hydroxyproline is determined following the guidelines of an ISO standard for meat and meat products —ISO 3496:1994—and that value is used to measure the collagen extraction yield, calculated as the ratio of extracted hydroxyproline in respect to its initial concentration in fish skin [41]. Besides, the content of hydroxyproline is also particularly relevant due to its relation to the thermal stability of collagen. It is known that hydroxyproline forms hydrogen bonds between collagen polypeptides, stabilizing the triple helix and thus a higher content of hydroxyproline would characterize a collagen exhibiting higher denaturation temperature [53]. More specifically, 4-Hyp residues increase the conformational stability of triple helix of collagen [54].

Nevertheless, more recently a complete analysis of the protein amino acids has been established as a standard study, allowing not only to evaluate the content of hydroxyproline, but also to assess the typical content of glycine representing about 1/3 of all amino acids in collagen. The amino acid analysis is composed by a protein hydrolysis followed by amino acid separation, identification and quantification using different chromatographic techniques [55]. The result is given as the number of moles of each amino acid in the sample, or as the number of residues of each amino acid per 1000 residues (the typical length of collagen alpha I chain) and since fish collagens (and marine collagens in general) are expected to have lower denaturation temperatures than their mammal counterparts, a lower hydroxyproline content in marine environment is presumed.

**Figure 3:**
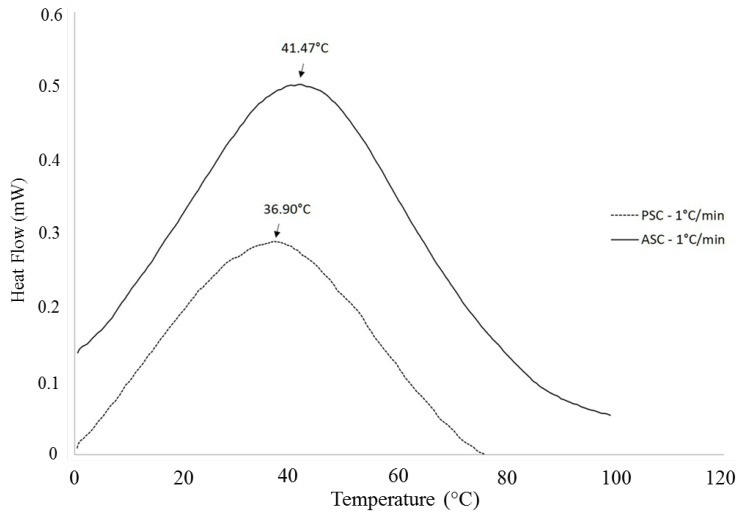
DSC thermogram of ASC and PSC extracted from the skin of codfish, obtained at a scan rate of 1 °C·min^−1^.

The thermal stability of collagen is attributed to the presence of amino acids like hydroxyproline, as abovementioned, and can be addressed by thermal analysis, namely differential scanning calorimetry (DSC) [56], by measuring the flux of calorimetric energy associated to material transitions, namely collagen denaturation, as a consequence of the heating process. As the heating process starts, protein absorbs heat, causing protein to unfold, at a temperature range specific to that protein, in this case, collagen [57]. The maximal transition temperatures and denaturation temperatures vary from different fish and, even if extracted from the same species, those temperatures are different if collagen is ASC or PSC [58]. The determination of denaturation temperature of ASC and PSC from the skin of codfish by DSC is illustrated in the thermograms in Figure 3. It should be noted that the scan rate at which the thermogram is obtained influences the determined value [59], by a process related with kinetics of denaturation [60] but not yet fully understood. It has been commonly described as an irreversible process, although some authors proposed a reversible process with an expressive hysteresis [61]. The denaturation temperature of the extracted collagen is a very important characteristic to account with, as it will influence the temperature that can be used in collagen processing into structures and its biomedical application.

**Figure 4:**
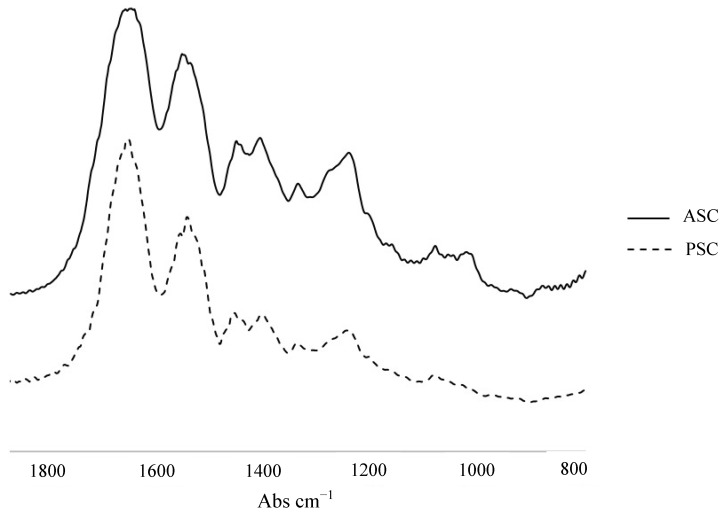
FTIR spectra of ASC and PSC extracted from skin of codfish, indicating the amide characteristic bands of collagen.

Fourier Transform Infrared Spectroscopy (FTIR) is a powerful tool to investigate and identify the chemical composition of a sample, like the presence of collagens and eventually their different types [62]. In the case in which collagen is extracted from a marine source, it is also used to compare the collagen composition obtained with and without the use of pepsin, to check if ASC and PSC are similar to each other [23,29,58], as exemplified by the spectra in Figure 4, exhibiting the characteristic amide peaks of collagens, observed in ASC and PSC extracted from the skins of codfish. It can also be used to check the presence of triple helix: if absorptions ratio of amide III (1235 cm^−1^) and the peak at 1450 cm^−1^ is close to 1, it is indicative that triple helix structure is preserved [63]. Also, according to Belbachir, K. *et al.* (2009), the classification of collagen types only by analysis of absorptions and infrared wavelength is not possible. However, applying a curve-fitting model to some spectra intervals, it presents a discrimination potential between studied collagen types and it can allow its classification [62].

Despite efforts focusing in other techniques, the most used to assess the helical content of a collagen sample is circular dichroism (CD), which addresses the structure of proteins. It uses the differences in the absorption of left- and right-handed polarized light by the sample as a function of the protein structural features [51].

To characterize morphological features, scanning electron microscope (SEM), transmission electron microscopy (TEM) and atomic force microscopy (AFM) are powerful tools for acquiring images of collagen samples, namely the presence and organization of collagen fibrils [64,65,66].

When considering its application, the level of different impurities and contaminants must also be addressed. In particular it is important to call attention to the assessment of the presence of sugars, heavy metals, endotoxins, among others, particularly critic when collagen is being considered for surgical implants, tissue engineering scaffolds and equivalent products, as stressed by the ASTM standard F 2212-11. Sugars content is determined following chromatographic methods, such as gas chromatography with mass spectrometry. By its turn, the presence of heavy metals can be addressed by atomic absorption spectroscopy or more appropriately by ion coupled plasma (ICP) techniques.

## 3. Applications

Collagen has been finding application in a myriad of fields and depending on the specific application and the desired formulation, different strategies can be followed to process collagen from marine origin. In principle, processing methodologies similar to the ones used with mammal collagens can be used with marine collagens, but due to specific characteristics of the latter, tuning of those methodologies may be needed and none should be taken for granted.

### 3.1. Tissue Engineering and Regeneration

Marine fish collagens are used in various biomedical applications [67]. Bone tissue engineering is the gold aim, with several studies in this field. As a recent example, Hoyer *et al.* [68] used salmon skin collagen with hydroxyapatite to produce scaffolds for bone regeneration purpose, based on biomimetic mineralization principle. Besides its mechanical elastic properties, it exhibited good absorption characteristics with interconnectivity between pores, which allowed human Mesenchymal Stem Cells (hMSCs) to adhere and proliferate, being a good base for osteogenic differentiation [68]. In the work of Nagai and co-workers [69], collagen extracted from salmon was cross-linked with 1-ethyl-3-(3-dimethylaminopropyl) carbodiimide hydrochloride (EDC) and also with dehydrothermal treatment (DHT) for periodontal regeneration applications. Terada *et al.* [70] developed a chitosan-collagen composite scaffold with oral mucosa regeneration purposes, using collagen extracted from tilapia scales.

Marine sponges are also an available source of collagen that has already proven to be comparable to vertebrate collagens. Heinemann *et al*. [71] used *C. reniformis*-derived collagen along with silica templating to produce hydrogels, which supported the adhesion, growth and differentiation of human mesenchymal stem cells into osteoblast cells. More studies have pointed that marine sponge collagen proved to be a good base for bone tissue engineering scaffolds [37]. Swatschek *et al.* [39] evaluated the use of marine sponge *C. reniformis* as a source of collagen to be integrated as a moisturizer in cosmetic preparations. Collagen isolated from the marine sponge *C. reniformis* was also used to prepare microparticles by emulsifying and cross-linking, which were then incorporated in hydrogels and the effects on drug-stability and on dermal drug delivery were evaluated [72]. Additionally, composites of chitosan, hydroxyapatite and collagen from *Ircinia fusca* have been also prepared using a freeze-drying methodology, where MG-63 osteoblast-like cells were able to adhere and proliferate, thus proposing such system for bone tissue repair [73].

Jellyfish collagen is also an available and relevant source to use as a matrix component for tissue engineering, since it exhibits low amount of impurities. Considering dry weight of edible jellyfish, more than 40% of it is collagen, in an animal where 95% of it is water [11]. As examples, porous scaffolds of jellyfish collagen from *Stomolophus nomurai meleagris* were prepared by freeze-drying and cross-linking with 1-ethyl-3-(3-dimethylaminopropyl) carbodiimide hydrochloride/*N*-hydroxysuccinimide (EDC/NHS) to be used in tissue engineering applications [49]. Jeong *et al.* [74] developed tubular scaffolds composed of jellyfish collagen and poly (d,l-lactide-*co*-glycolide acid) (PLGA), by freeze-drying and electrospinning, proposing their application on vascular grafts. By their turn, Addad *et al.* [50] proposed the use of jellyfish collagen cross-linked with EDC-NHS, at different ratios, as substitution of bovine or human collagens in different biomedical applications. More recently, porous scaffolds composed by refibrillized collagen, previously extracted from jellyfish *Rhopilema esculentum*, showed an elastic behavior and were capable of supporting the culture of hMSCs, being observed the overexpression of chondrogenic markers under chondrogenic stimulation, suggesting the use of such structures as scaffolds for cartilage tissue engineering [75]. Besides, collagen extracted from other jellyfish species have also been proposed for further use in various applications, as is the case of the ones extracted from *Cyanea nozakii* [76], one of the jellyfish species forming large blooms in Chinese seas [77].

Collagen from marine gastropods has been proposed as well, in particular the ones extracted from different body parts of *Ficus variegate*, for the development of porous structures upon crosslinking with several agents, including chitosan, hydroxyapatite and glutaraldehyde, to be further used in different biomedical applications [78].

Although marine collagen is considered to possess low antigenicity [79] when considering its use in Tissue Engineering and Regeneration applications, *in vivo* studies should be performed to study the viability of using the chosen collagen in human implants [80,81].

### 3.2. Cosmetic, Skin Care and Other Medical Applications

Collagens have been recognized for their biological action, with a great potential to be used in the cosmetic field, where new generations bring new targets, toward beauty and maintenance of young appearance, and the search for safe and inexpensive ingredients are constant. In this regard, marine proteins, and marine collagens in particular, nowadays, are being presented as excellent functional ingredients for the cosmetic industry [17,82,83,84]. For instance, its properties take to the development of creams and gels with high moisturizing action, but other activities are also foreseen, as anti-aging, anti-wrinkling or UV radiation protectors [85]. For the cosmetic industry, marine collagens have been obtained from coldwater fish skins, such as cod, haddock and salmon [86,87]; besides, it is also produced from fish scales, through decalcification and enzymatic hydrolysis [88]. 

Marine collagens has been found to have applications in the healing of wounds resulting from different traumas (burns, grafting, ulcerations, *etc*.), with collagen-based materials being used mainly to prevent moisture and heat loss from the wounded tissue, while providing as well a microbial infiltration barrier [82,83]. Besides, they have also been used in drug delivery systems, for example, collagen shields in ophthalmology, mini-pellets and tablets for protein delivery, gel formulation in combination with liposomes as controlling material for transdermal delivery, and nanoparticles for gene delivery [72,82,83,89,90,91].

## 4. Future Perspectives

This review should lead us to better assess or re-assess the commercial potential alternatives of collagens produced from marine sources.

Collagens may not be so easily and advantageously replaced with other molecules, in their current commercial and/or under development compositions as economically viable options and/or with similar biocompatibility profiles and physic-chemical and biological properties. But, they also present some risks due to their human or animal origins.

Recent major outbreaks of transmissible diseases, such as prion diseases (e.g., bovine spongiform encephalopathy (mad cow disease), Creutzfeld-Jacob disease), severely impacted bovine and human derived healthcare products, with reduced usage—even prohibited for some indications (e.g., human derived products for spinal cord and brain surgeries)—manufacturing shutdowns, change of animal sources, strengthened regulatory approval barriers (as illustrated in the following references and many others: [92,93,94,95,96]. Even if the residual risk of transmissible diseases by bovine and human derived healthcare products can be reduced to zero or at an extremely low level, these products may still look suspicious by end consumers.

Other major issues come from cultural practices and religious beliefs, which may seriously restrain the use of bovine and porcine derived products by some consumers and in world regions. This has been extensively presented and discussed in recent reviews [97,98,99].

Elegant and attractive alternative sources of collagens have been developed and proposed, by using recombinant expression techniques. This allowed the expression of several human collagen types in bioreactor-based eukaryotic systems and their subsequent isolation and purification [100]. The yeast *Pichia pastoris* has been the preferred vector for the recombinant expression of human collagens [101,102,103], but this genetically engineered process has been successful in plant cells as well [104,105,106]. Nevertheless, the production of human recombinant collagens is rather complex. In particular, it relies on the co-expression of collagen genes with prolyl 4-hydroxylase genes for obtaining thermally stable collagens. Besides, the economic viability of human recombinant collagens is highly challenging and strongly dependent on high accumulation of properly assembled collagens coupled with the ability to grow the cell expression system at a high cell density [100]. So far, even if clinical programs with human recombinant collagen derived products successfully ran and showed their very good tolerance and biocompatibility, it is believed that they are still only commercially available to research applications.

Marine collagens should be a true alternative source of collagens. Marine species present a distinct advantage as a lower known risk of transmission to humans of infection-causing agents [107,108] and are thought to be far less associated with cultural and religious concerns regarding the human use of marine derived products.

As discussed in the previous sections of the present review, the “standard” types of collagens, including type I collagen widely used in healthcare products and foods, have been isolated and purified from several mammal and non-mammal marine sources, at a high purity and with satisfying yields, by using conventional collagen purification techniques. But, when native collagens, with non-denatured triple helix, are intended to be used in human healthcare products, they should be, however, selected among those displaying the higher denaturation temperatures, such as shark collagens. It is also anticipated that it may be requested an additional structural stabilization of such marine collagens by chemical derivatizations, resulting in higher denaturation temperatures and increased resistance to enzymatic degradation [109].

What lacks the most for the commercial exploitation of marine collagens, from the available literature, particularly as components of healthcare products? Industrial translation of the manufacturing processes, pondering the following five main aspects, as regulated by safety and risk management standards and laws, applicable to food ingredients, cosmetics, medical devices and drugs, with examples of such references for medical devices.

—*Sustainability of the marine source*. The source should be available for years without major supply disruptions, at sufficient quantities. It should allow the manufacturing of at least >10 kg pure collagen each year—more preferably >100 kg each year—for any worldwide distributed healthcare product and more than several tons each as a component of food products, as deduced from collagen derived product market [110].

—*Safety profile*. Marine collagens should be as safe as their animal origin counterparts. As components of medical devices or pharmacological products, they should pass all the biocompatibility tests, such as for medical devices, ISO 10993 tests [111], in accordance to the Essential Requirements defined in the Annex I of the COUNCIL DIRECTIVE 93/42/EEC [112], required by the corresponding international/national regulations. In particular, they should present acceptable immunotoxicity and allergy/anaphylaxis risks, which may be challenging due to large phylogeny differences between human and marine species, in particular non-mammal marine species.

—*Residues/Chemical and biological pathogens*. The manufacturing process of marine collagens should be fully validated and be robust enough for the reproducible and consistent removal of any pathogens/residues, potentially harmful in humans. This is driven by safety regulations, including for example, for medical devices, the risk management standard ISO 14971 [113] and in Europe, the COUNCIL DIRECTIVE 93/42/EEC, defining the Essential Requirements [112]. In particular, it should pass microbial challenges with known relevant marine microorganisms, *i.e.*, elimination by a factor >10^6^ of known loads of marine viruses or bacteria, added in the raw product or at intermediate steps of the manufacturing process, identified as removing pathogens.

—*Reproducibility of marine collagen batches*. Marine collagen batches must be designed and manufactured in such a way as to guarantee the characteristics and performances (for example, for medical devices, in Europe, see Annex I of [112]). They should relentlessly pass pre-defined specifications of a battery of tests, guaranteeing the identity and purity of collagens, such as non-exhaustively, hydroxyproline analysis, SDS Page electrophoresis profile (e.g., collagen identity/(sub)type, molecular weight, hydrolysis products, and chain distribution), Differential Scanning Calorimetry (e.g., denaturation temperature and enthalpy), heavy metals, carbohydrates (e.g., glycosylation products). The manufacturing process should be robust enough to show batch-to-batch consistency, *i.e.*, giving the same anticipated/expected batch-to-batch quality and amount/yield of collagens.

—*Industrial batches: economic viability*. Each collagen batches should conveniently be larger than 1 kg for healthcare products and much larger for food applications. The target standard cost of marine collagens is believed to be in the <100 USD per kilogram range for food applications and 5–50 USD range per gram for healthcare products. These assumptions are deduced by the average selling prices of collagen-based products (see for example [110]).

Before investing time and money in a marine collagen project, there are also other questions that must be placed, besides the most technical ones. The potential adoption curve for marine collagen based products should also be carefully considered, their market potential, the intellectual property around them, their competition, the overall project costs (e.g., implementation of manufacturing lines, development of marine collagen derived products), timing considerations (e.g., handoffs, funding, regulatory, marketing, *etc*.), the business climate (e.g., economic, societal, regulatory, liability, image, morale, *etc*.), exit, anticipation or survival Strategy [114].

But, the stakes of marine collagens are very high. Marine collagens should offer new and real opportunities for first to market options, particularly in healthcare, diversification, and lowering the safety risks and cultural and religious concerns, by developing know-how of collagen manufacturing from different sources.

It is certainly a risk paying off (when is the question), but scientifically there are no doubts that it is a route that deserves to be taken.
